# Editorial: Intracellular Molecular Processes Affected by pH

**DOI:** 10.3389/fmolb.2022.891533

**Published:** 2022-04-11

**Authors:** Jan M. Antosiewicz, Patricia M. Kane

**Affiliations:** ^1^ Faculty of Physics, Institute of Experimental Physics, University of Warsaw, Warsaw, Poland; ^2^ Department of Biochemistry and Molecular Biology, SUNY Upstate Medical University, Syracuse, NY, United States

**Keywords:** protons and pH inside cells, modelling molecular pH sensing, maintaince of cellular pH, pH dependent intracellular protein localization, mechanisms of pH dependence of viral infection

pH influences molecular processes when the molecules involved are capable of proton exchange with the solvent and/or other molecules. In living systems, pH impacts function at every level, from the structure and interactions of individual molecules to the function of organelles and the overall organization of the cell. Advances over the past 2 decades have provided the ability to measure and control pH in nano-scale environments, experimental access to single molecules *in vitro* and *in vivo*, and improved computational modeling of pH-dependent molecular mechanics and dynamics. Together, these techniques are elucidating the complex roles of pH in living systems.

The behavior of protons in aqueous solution is at the root of various acid-base equilibria of functional groups in biomolecules and the impact of pH of structure, dynamics, and function. Silverstein discusses models for hydrated proton structure and relates these structures to proton mobility in different environments. These principles are applied to illustrate the influence of pH on protein structure, enzyme kinetics, and biochemical thermodynamics. They also affect how pH is viewed in the context of intact cells.

pH-dependent biological processes are determined by acid-base properties of participating molecules. Warwicker describes the properties of proteins known to exhibit pH-dependent sensing and function and discusses how these properties can be incorporated into predictive computational modeling of pH-dependent molecular structure. This approach will ultimately allow prediction of pH dependent properties in the wealth of proteomic and structure data currently emerging.

Eukaryotic cells maintain a cytosol of near-neutral pH surrounding both acidic and alkaline organelles. Doyen et al. discuss the importance and challenges of accurately measuring cytosolic and organelle pH. Maintenance of organelle and cytosolic pH balance poses a major challenge for cells, and they outline the major players in pH homeostasis. Although mathematical modeling suggests minimal set of plasma membrane transporters could maintain cytosolic pH, they propose that the biochemical complexity and apparent redundancy in transporters may be required to protect cells from intracellular and environmental challenges to pH homeostasis.

Viruses exploit pH-dependent conformational changes to support infection. As described by Caffrey and Lavie, influenza hemagglutinin undergoes dramatic conformational changes at low endosomal pH. These pH-triggered changes provide both a mechanism of infection and an opportunity for designing therapeutics. Warwicker predicts pH-switching between conformational forms in the SARS-CoV2 spike protein and its variants.

Tokmakov et al. view the related properties of protein pH and pI (isoelectric point) from the perspective of entire cell proteomes. Intriguingly, a protein’s subcellular localization correlates with its pI across multiple organisms, suggesting that proteins have evolved to match their properties to the pH of their organelle or membrane environment.

This collection samples the global importance of pH in cellular and molecular biology but is the tip of an enormous iceberg. Improvements in experimental and computational methods will provide a deeper understanding of the biophysical basis of pH sensing and function in biomolecules. Measurement of simultaneous real-time pH changes in multiple cellular locales will enhance our understanding of cellular pH balance. Together these methods will elucidate the multiple layers of pH control in biological systems.

